# Transcranial direct current stimulation (tDCS) for upper limb rehabilitation after stroke: future directions.

**DOI:** 10.1186/s12984-018-0459-7

**Published:** 2018-11-15

**Authors:** Bernhard Elsner, Joachim Kugler, Jan Mehrholz

**Affiliations:** 1 Department of Public Health, Dresden Medical School, Technical University Dresden, Fetscherstr. 74, 01307 Dresden, Germany; 2 SRH University of Applied Health Sciences, Gera, Germany

**Keywords:** Stroke, Arm, Rehabilitation, Transcranial direct current stimulation, Magnetic resonance imaging, Electroencephalography

## Abstract

Transcranial Direct Current Stimulation (tDCS) is a potentially useful tool to improve upper limb rehabilitation outcomes after stroke, although its effects in this regard have shown to be limited so far. Additional increases in effectiveness of tDCS in upper limb rehabilitation after stroke may for example be achieved by (1) applying a more focal stimulation approach like high definition tDCS (HD-tDCS), (2) involving functional imaging techniques during stimulation to identify target areas more exactly, (3) applying tDCS during Electroencephalography (EEG) (EEG-tDCS), (4) focusing on an effective upper limb rehabilitation strategy as an effective base treatment after stroke. Perhaps going even beyond the application of tDCS and applying alternative stimulation techniques such as transcranial Alternating Current Stimulation (tACS) or transcranial Random Noise Stimulation (tRNS) will further increase effectiveness of upper limb rehabilitation after stroke.

## Background

Impaired arm function after stroke is both frequent and a considerable burden for people with stroke and their caregivers. An emerging approach for enhancing neural plasticity after acute and chronic brain damage, thus enhancing rehabilitation outcomes in the upper limb rehabilitation after stroke, is non-invasive brain stimulation (NIBS), for example delivered by transcranial direct current stimulation (tDCS) [1]. tDCS is a potentially useful tool for facilitating neural plasticity, because it is relatively inexpensive, easy to administer and safe.

Many small trials regarding the effects of tDCS on arm motor function poststroke were undertaken in the past with partly promising but not conclusive results [2, 3]. Based on these trials a lot of research interest increased in the last 10 to 15 years which still persists. This considerable research interest is a bit surprising first, given the fact that this type of therapy is not used across the board in clinical routine and second, the largest multicenter randomized clinical trial with appropriate methodology including 96 patients did not find clear results in favor of this type of stimulation [4]. A recent network meta-analysis of randomised controlled trials about the effectiveness of tDCS suggested only limited evidence for effectiveness of tDCS after stroke for arm rehabilitation [3]. The optimal stimulation paradigm regarding polarisation, electrode location, amount of direct current applied and stimulation duration still has to be established in order to maximize clinical effectiveness of tDCS [5]. Additionally, doubts emerged that the underlying rationale, the interhemispheric competition model, may be oversimplified or even incorrect [6]. The interhemispheric competition model postulates that a stroke leads to an inhibition of the ipsilateral and to an (over-) excitation of the contralateral brain hemisphere. Hence its clinical implications are to inhibit the contralateral hemisphere and to excited ipsilateral hemisphere. Moreover, electrode positioning and the resulting direction of electric fields as well as variation in head anatomy also modulate stimulation effects [7, 8]. Hence, further approaches may be warranted beyond the approach of neuronavigation prior to stimulation: Additional increases in effectiveness of tDCS in upper limb rehabilitation after stroke may for example be achieved by (1) applying a more focal stimulation approach like high definition tDCS (HD-tDCS), (2) involving functional imaging techniques during stimulation to identify target areas more exactly, (3) applying tDCS during EEG (EEG-tDCS), (4) focusing on an effective upper limb rehabilitation strategy as an effective base treatment after stroke. Perhaps going even beyond the application of tDCS and applying alternative stimulation techniques such as transcranial Alternating Current Stimulation (tACS) [9] or transcranial Random Noise Stimulation (tRNS) [10] will further increase effectiveness of upper limb rehabilitation after stroke.

## Future directions

### High definition (HD)-tDCS

The problem of limited focal specificity of tDCS may lead to an ineffective stimulation and in turn may be reduced by the application of HD-tDCS, which involves up to five small electrodes, arranged in a ring, instead of two big conventional sponge electrodes for delivering direct current. The positioning of HD-tDCS for improving arm motor function after stroke has been described elsewhere. [11] On the one hand side, the increased focality of this electrode setup has been proven by mathematical modelling of electrical current flow based on MRI acquired individual tissue volumina and their corresponding electrical conductivity. [11] On the other hand side, increased focality of stimulation may be more sensitive to electrode misplacement. To our knowledge, there are no randomised controlled trials of the effects of HD-tDCS on arm rehabilitation after stroke. Richardson et al. performed a randomised controlled trial of HD-tDCS in treating people with aphasia after stroke suggested that HD-tDCS is feasible, likely to be acceptable to both, patients and clinicians and not inferior to conventional tDCS [12]. So one could argue that the use of HD-tDCS could improve upper limb rehabilitation outcomes after stroke by improving focality of stimulation.

### TDCS during functional magnetic resonance imaging (fMRI)

TDCS during fMRI has already successfully been applied in language production tasks in healthy people and in people with stroke [13]. Meinzer and colleagues used an MRI-compatible tDCS device, with additional filter boxes and resistor-mounted electric cables. This special setup is necessary to avoid interferences between the magnetic fields induced by the fMRI on the one hand and the tDCS stimulator on the other hand. Yet the tDCS stimulator has to be operated from outside the MRI scan room. The authors emphasised the need to pay attention to the fMRI and tDCS-specific contraindications and to perform image quality assurance before scanning, in order to check for tDCS-induced artefacts [14]. During active tDCS they first performed a resting state fMRI for five minutes, followed by a semantic word generation task for 11 min, completed by additional structural scans. This combined application of tDCS with concurrent fMRI creates the possibility to examine the underlying neurophysiological effects of tDCS with high spatial resolution and hence to estimate the most promising location for electrode placement. It also eases the process of identifying tDCS-responders and non-responders. A considerable challenge in exploring this approach to upper limb rehabilitation research after stroke is the adaptation of arm rehabilitation strategies or devices to the spatial and safety restrictions in the standard fMRI scanner. One possibility to overcome the spatial restrictions to some extent could be the use of an open fMRI scanner. So tDCS during fMRI for determining brain areas of interest may be another approach for increasing tDCS effectiveness also in upper limb rehabilitation after stroke. To our knowledge, there are no published trials examining the effects of tDCS by fMRI for improving outcomes in upper limb rehabilitation post stroke so far.

### EEG-tDCS

Based on EEG data, it is possible to measure cortical activity after or even during tDCS with superior temporal resolution [15]. There are devices which integrate tDCS and EEG electrodes in a single head cap which is then connected to a computer for controlling stimulation and recording and analysing EEG signals, simultaneously. Miniussi and colleagues concluded that the latter approach of online EEG and tDCS could gain particularly valuable insights into the effects of brain stimulation on widespread cortical network function. Outcome measures of interest are acute and persistent changes in spontaneous neural activity on the delta, theta and alpha band and event -related the desynchronisation/synchronisation (ERD/ERS) in the alpha and beta bands [16]. ERD/ERS are biomarkers in motor rehabilitation. [17] There also is the possibility of invasive recordings with intracranial EEG-electrodes (intracranial subdural strips or intracranial depth electrodes), which deliver a signal with fewer artefacts [18]. To our knowledge, there are no published trials examining the effects of tDCS for improving outcomes in upper limb rehabilitation post stroke as measured by EEG so far.

### Effective base treatment (arm rehabilitation)

Since tDCS enhances neuroplasticity by modulating cortical activity, the application of an efficient base therapy, resulting in sufficient cortical activity, is still crucial. Therefore is has to be kept in mind that tDCS has to be applied in combination with an appropriate and individually selected and effective arm training: For instance in people with severe arm paresis it makes sense to apply electromechanically-assisted arm training whereas in people with only minor arm paresis a forced use paradigm seems to be promising to improve arm function and to overcome learned non use. There is a considerable evidence base regarding effective base treatments during tDCS for improving upper limb rehabilitation outcomes poststroke.

### Alternative stimulations or applications

An alternative to tDCS could be the application of transcranial Alternating Current Stimulation (tACS) to increase excitability [9]. tACS aims at stimulating neuronal oscillations between 80 and 200 Hz. Different protocols exists, for instance a tACS protocol of 140 Hz delivered at 1 mA for 10 min might produce after-effects comparable in duration to those induced by anodal tDCS [9].

Another recent non-invasive approach to brain stimulation is transcranial Random Noise Stimulation (tRNS) [2]. tRNS delivers electrical current in the “noise” -mode, which means that the flow of current consistently and randomly changes between − 1000 μA and 1000 μA with a mean of 0 μA. It uses frequencies ranging from 0.1 to 640 Hz and may lead to consistent increase in motor cortex excitability by repeated potentiation of sodium channels [10].

## Conclusion

The mode of action of tDCS is not fully understood yet, which has implications for its clinical application: for example, tDCS suffers from a lack of focality of stimulation, and attaining neurophysiological data in adequate spatial and temporal resolution for further exploration of its mode of action remains challenging. HD-tDCS may be a one tool for reducing variability in future tDCS trials in upper limb rehabilitation after stroke by increasing focality of stimulation. Together with tDCS during fMRI, which allows for superior spatial resolution, online EEG-tDCS delivering superior temporal resolution could give valuable insights into tDCS mechanisms. Hopefully, this will improve our understanding of tDCS in upper limb rehabilitation in people with stroke and corollary also its clinical application. However, with tDCS being rather a facilitatory intervention thus needing an effective base treatment resulting in sufficient neuroplasticity, it requires efficient interventions delivered by therapists (e.g. forced use, electromechanical-assisted arm training for the more severely affected patients).

Particularly from the perspective of clinicians, it eventually should be kept in mind that despite the currently considerable research interest in tDCS, it is still likely that tDCS reveals only small or even no additional benefits to arm rehabilitation after stroke.

## References

[1] Priori A, Berardelli A, Rona S, Accornero N, Manfredi M (1998). Polarization of the human motor cortex through the scalp. Neuroreport.

[2] Lefaucheur JP, Antal A, Ayache SS, Benninger DH, Brunelin J, Cogiamanian F, Cotelli M, De Ridder D, Ferrucci R, Langguth B (2017). Evidence-based guidelines on the therapeutic use of transcranial direct current stimulation (tDCS). Clin Neurophysiol.

[3] Elsner B, Kwakkel G, Kugler J, Mehrholz J (2017). Transcranial direct current stimulation (tDCS) for improving capacity in activities and arm function after stroke: a network meta-analysis of randomised controlled trials. J Neuroeng Rehabil.

[4] Hesse S, Waldner A, Mehrholz J, Tomelleri C, Pohl M, Werner C (2011). Combined transcranial direct current stimulation and robotassisted arm training in subacute stroke patients: a randomized, double-blind multicenter study. Neurorehabil Neural Repair.

[5] Floel A (2014). TDCS-enhanced motor and cognitive function in neurological diseases. Neuroimage.

[6] Di Pino G, Pellegrino G, Assenza G, Capone F, Ferreri F, Formica D, Ranieri F, Tombini M, Ziemann U, Rothwell JC, Di Lazzaro V (2014). Modulation of brain plasticity in stroke: a novel model for neurorehabilitation. Nat Rev Neurol.

[7] Rampersad SM, Janssen AM, Lucka F, Aydin U, Lanfer B, Lew S, Wolters CH, Stegeman DF, Oostendorp TF (2014). Simulating transcranial direct current stimulation with a detailed anisotropic human head model. IEEE Trans Neural Syst Rehabil Eng.

[8] Datta A, Truong D, Minhas P, Parra LC, Bikson M (2012). Inter-individual variation during transcranial direct current stimulation and normalization of dose using MRI-derived computational models. Front Psychiatry.

[9] Moliadze V, Antal A, Paulus W (2010). Boosting brain excitability by transcranial high frequency stimulation in the ripple range. J Physiol.

[10] Terney D, Chaieb L, Moliadze V, Antal A, Paulus W (2008). Increasing human brain excitability by transcranial high-frequency random noise stimulation. J Neurosci.

[11] Datta A, Bansal V, Diaz J, Patel J, Reato D, Bikson M (2009). Gyri-precise head model of transcranial direct current stimulation: improved spatial focality using a ring electrode versus conventional rectangular pad. Brain Stimul.

[12] Richardson J, Datta A, Dmochowski J, Parra LC, Fridriksson J (2015). Feasibility of using high-definition transcranial direct current stimulation (HD-tDCS) to enhance treatment outcomes in persons with aphasia. NeuroRehabilitation.

[13] Meinzer M, Lindenberg R, Darkow R, Ulm L, Copland D, Floel A. Transcranial direct current stimulation and simultaneous functional magnetic resonance imaging. J Vis Exp. 2014;86:e51730. 10.3791/51730 PMC4181345 24796646

[14] Antal A, Bikson M, Datta A, Lafon B, Dechent P, Parra LC, Paulus W (2014). Imaging artifacts induced by electrical stimulation during conventional fMRI of the brain. Neuroimage.

[15] Miniussi C, Brignani D, Pellicciari MC (2012). Combining transcranial electrical stimulation with electroencephalography: a multimodal approach. Clin EEG Neurosci.

[16] Roy A, Baxter B, He B (2014). High-definition transcranial direct current stimulation induces both acute and persistent changes in broadband cortical synchronization: a simultaneous tDCS-EEG study. IEEE Trans Biomed Eng.

[17] Molteni E, Cimolin V, Preatoni E, Rodano R, Galli M, Bianchi AM (2012). Towards a biomarker of motor adaptation: integration of kinematic and neural factors. IEEE Trans Neural Syst Rehabil Eng.

[18] Taussig D, Montavont A, Isnard J (2015). Invasive EEG explorations. Neurophysiol Clin.

